# Antibacterial Co(II) and Ni(II) Complexes of *N*-(2-Furanylmethylene)-2-Aminothiadiazole and Role of SO_4_^2−^, NO_3_^−^, C_2_O_4_^2−^ and CH_3_CO_2_^−^ anions on Biological Properties

**DOI:** 10.1155/MBD.2002.287

**Published:** 2002

**Authors:** Zahid H. Chohan, Abdul Rauf, Claudiu T. Supuran

**Affiliations:** 1 Department of Chemistry Islamia University Bahawalpur Pakistan; 2 University of Florence Dipartimento di Chimica Laboratorio di Chimica Bioinorganica Via della Lastruccia 3, Rm 188, Polo Scientifico Firenze 50019-Sesto Fiorentino Italy

## Abstract

Co(II) and Ni(II) complexes with a Schiff base, *N*-(2-furanylmethylene)-2-aminothiadiazole have been prepared and characterized by their physical, spectral and analytical data. The synthesized Schiff-bases act as tridentate ligands during the complexation reaction with Co(II) and Ni(II. metal ions. They possess the composition [M(L)_2_]X_n_ (where M=Co(II) or Ni(II), L=, X=NO_3_^−^, SO_4_^2−^, C_2_O_4_^2−^ or CH_3_CO_2_^−^ and n=1 or 2) and show an octahedral geometry. In order to evaluate the effect of anions upon chelation, the Schiff-base and its complexes have been screened for antibacterial activity against bacterial strains e.g., *Escherichia coli*, *Staphylococcus aureus*, and *Pseudomonas aeruginosa*.


### ANTIBACTERIAL Co(II) AND Ni(II) COMPLEXES OF *N*-(2-FURANYLMETHYLENE)-2-AMINOTHIADIAZOLE AND ROLE OF $SO_4^{2-}$, $NO_3^-$, $C_2O_4^{2-}$ AND $CH_3CO_2^-$ ANIONS ON BIOLOGICAL PROPERTIES

Zahid H. Chohan*[1], Abdul Rauf[1] and Claudiu T. Supuran[2]

[1] Department of Chemistry, Islamia University, Bahawalpur, Pakistan
[2] University of Florence, Dipartimento di Chimica, Laboratorio di Chimica Bioinorganica, Via della Lastruccia 3, Rm 188, Polo Scientifico, 50019-Sesto Fiorentino Firenze, Italy

### ABSTRACT
Co(II) and Ni(II) complexes with a Schiff base, *N*-(2-furanylmethylene)-2-aminothiadiazole have been prepared and characterized by their physical, spectral and analytical data. The synthesized Schiff-bases act as tridentate ligands during the complexation reaction with Co(II) and Ni(II) metal ions. They possess the composition $[M(L)_2]X_n$ (where M=Co(II) or Ni(II), L=, X=$NO_3^-$, $SO_4^{2-}$, $C_2O_4^{2-}$ or $CH_3CO_2^-$ and n=1 or 2) and show an octahedral geometry. In order to evaluate the effect of anions upon chelation, the Schiff-base and its complexes have been screened for antibacterial activity against bacterial strains e.g., *Escherichia coli*, *Staphylococcus aureus*, and *Pseudomonas aeruginosa*.

### INTRODUCTION
Many studies[1-3] have indicated the relationship between the metal ions and their complexes as antitumour[4-9] and antibacterial[10,11] agents. *In vivo* tests have shown that biologically active compounds become more carcinostatic and bacteriostatic upon chelation[16]. Such interactions with metal ions, particularly those of the transition metal ions with amino acids, peptides and nucleic acids, are of relevant biological importance[13-15]. Several reviews[16-19] have shown that the coordination of such compounds with metal ions markedly influence their biological action highlighting not only their catalytic function[20-23] but the nature of their chemical action[24-27] as well. Many potent antibacterial and antifungal compounds are reported[28,29] by condensation of salicylaldehyde with heterocyclic compounds. Thiadiazole derived compounds of salicylaldehyde were found[30] to be bactericidal/fungicidal against *Bacillus cereus* and *Aspergillus niger*. Several others, incorporating piperazinyl guanidines, when condensed with salicylaldehyde were found to exhibit cardiovascular and vasodepressor activity.[31] Similarly, thiazolidinone-derived salicylaldehydes[32] possess antimicrobial activity against many pathogenic strains. Keeping in view the significance of thiadiazole or its derived compounds and the role of metals in biology, we have previously reported[33] series of biologically active compounds and have evaluated the role of metal ions on their activity. In continuation to the same, we have prepared several other biologically active cobalt(II) and nickel(II) complexes of the type $[M(L)_2]X_n$ where M=Co(II), Ni(II) or Zn(II), L=*N*-(2-furanylmethylene)-2-aminothiadiazole (Fig 1), X= $NO_3^-$, $SO_4^{2-}$, $C_2O_4^{2-}$ or $CH_3CO_2^-$ and n=1 or 2, having the same metal ion (cation) but, different anions and wish to report the possible biological role of anions against bacterial strains of *Escherichia coli*, *Staphylococcus aureus*, and *Pseudomonas aeruginosa*.

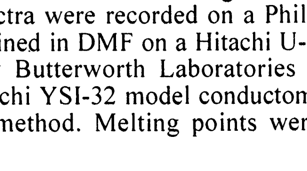

Fig 1. Structure of the Schiff base (L)

### EXPERIMENTAL
### Material and Methods
All chemicals and solvents used were of Analar grade. The metal(II) salts were used as nitrates, sulfates, oxalates and acetates. IR spectra were recorded on a Philips Analytical PU 9800 FTIR spectrophotometer. UV-Visible spectra were obtained in DMF on a Hitachi U-2000 double-beam spectrophotometer. C, H and N analyses was carried out by Butterworth Laboratories Ltd. Conductance of the metal complexes was determined in DMF on a Hitachi YSI-32 model conductometer. Magnetic measurements were made on solid complexes using the Gouy method. Melting points were recorded on a Gallenkamp apparatus and are uncorrected.

**Preparation of *N*-(furanylmethylene)-2-aminothiadiazole (L)**

It was prepared[34] and characterized by the same method reported earlier.

**Preparation of Metal(II) Complex.**

A warm ethanol solution (20 mL) of the respective Schiff base (0.002 M) was added to a magnetically stirred solution of the metal(II) salt (0.001 M) in ethanol (25 mL). The mixture was refluxed for 1 h and cooled to room temperature. On cooling, precipitates were formed which were filtered, washed with ethanol, acetone and ether, and dried. Crystallization in aqueous ethanol (30:70) gave the desired metal complex. All other metal complexes were prepared respectively following the same method.

**Antibacterial Studies**

The synthesized metal complexes, in comparison to the uncomplexed Schiff-base ligands were screened for their antibacterial activity against pathogenic bacterial species, *Escherichia coli, Staphylococcus aureus* and *Pseudomonas aeruginosa*. The paper disc diffusion method[33] was adopted for the determination of antibacterial activity.

### RESULTS AND DISCUSSION

**Physical Properties**

The Schiff-base (Fig. 1) was prepared[34] by refluxing an appropriate amount of 2-amino-1,3,4-thiadiazole and furan-2-carboxaldehyde in ethanol in a 1:1 molar ratio. It was further used for complexation with the Co(II) and Ni(II) metal ions. All the newly synthesized metal complexes (Table 1) were prepared by the stoichiometric reaction of the corresponding metal salts as their nitrate, sulfate, acetate and oxalate and the corresponding Schiff-base in a molar ratio M:L of 1:2 as shown by Scheme 1.

$$MX_n \quad + \quad 2L \longrightarrow [M(L)_2](X)_n$$

M=Co(II) or Ni(II)

L=Fig.1

X=$SO_4^{2-}$, $NO_3^-$, $C_2O_4^{2-}$ or $CH_3CO_2^-$

n=1 or 2

(Scheme 1)

These complexes are air and moisture stable, intensely colored, amorphous solids that decompose above 200° C. They are insoluble in common organic solvents like ethanol, methanol, chloroform or acetone but soluble in DMSO and DMF. Molar conductance values of the soluble complexes in DMF show their values (142-148 $ohm^{-1}cm^2mol^{-1}$) indicating[35] that they are all electrolytic in nature.

Table 1. Physical and Analytical Data of the Metal(II) Complexes

| No | Metal chelate/ Mol. Formula | Yield (%) | M.P (°C) (decomp) | B.M. ($\mu_{eff}$) | Calc (Found)% C H N |
|---|---|---|---|---|---|
| (1) | $[Co(L)_2](SO_4)$ [512.9] $C_{14}H_{10}CoN_6O_6S_3$ | 63 | 211-213 | 4.6 | 32.8 1.9 16.4 (33.2) (1.7) (16.5) |
| (2) | $[Co(L)_2](NO_3)_2$ [540.9] $C_{14}H_{10}CoN_8O_8S_2$ | 62 | 218-220 | 4.7 | 31.1 .1.8 20.7 (31.4) (2.0) (20.5) |
| (3) | $[Co(L)_2](C_2O_4)$ [504.9] $C_{16}H_{10}CoN_6O_6S_2$ | 61 | 209-211 | 4.6 | 38.0 2.0 16.6 (38.2) (2.1) (16.9) |
| (4) | $[Co(L)_2](CH_3CO_2)_2$ [534.9] $C_{18}H_{16}CoN_6O_6S_2$ | 63 | 215-217 | 4.7 | 40.4 3.0 15.7 (40.6) (2.8) (15.9) |
| (5) | $[Ni(L)_2](SO_4)$ [512.7] $C_{14}H_{10}NiN_6O_6S_3$ | 62 | 215-217 | 3.3 | 32.8 2.0 16.4 (32.5) (2.4) (16.3) |
| (6) | $[Ni(L)_2](NO_3)_2]$ [540.7] $C_{14}H_{10}NiN_6O_6S_3$ | 62 | 220-222 | 3.4 | 31.0 1.8 20.7 (30.8) (1.6) (20.4) |
| (7) | $[Ni(L)_2](C_2O_4)$ [504.7] $C_{14}H_{10}NiN_6O_6S_3$ | 60 | 211-213 | 3.5 | 38.0 2.0 16.6 (38.2) (2.3) (16.9) |
| (8) | $[Ni(L)_2](CH_3CO_2)_2$ [534.7] $C_{14}H_{10}NiN_6O_6S_3$ | 61 | 216-218 | 3.3 | 40.4 3.0 15.7 (40.5) (2.8) (15.5) |

**Infrared Spectra**

IR spectra of the Schiff-base showed the absence of bands at 1735 and 3420 $cm^{-1}$ due to carbonyl ν(C=O) and ν($NH_2$) stretching vibrations and, instead, appearance of a strong new band at ~1635 $cm^{-1}$ assigned[36] to the azomethine ν(HC=N) linkage. It suggested that amino and aldehyde moieties of the starting reagents are absent and have been converted into the azomethine moiety (Fig.1). The comparison of the IR spectra of the

Schiff-base and its metal chelates (Table 2) indicated that the Schiff-base was coordinated to the metal atom in three ways, representing them acting in a tridentate manner. The band appearing at 1625 $cm^{-1}$ due to the azomethine was shifted to lower frequency by ~10-15 $cm^{-1}$ indicating[37] participation of the azomethine nitrogen in complexation. The band at 1610 assigned to thiadiazole ring ν(C=N) nitrogen also shifted to lower frequency by ~15-25 $cm^{-1}$ that was also indicative of the involvement of ring nitrogen of thiadiazole in chelation.

Further conclusive evidence of the coordination of the Schiff-base with the metals, was shown by the appearance of weak low frequency new bands at 525-530 and 455-460 $cm^{-1}$. These were assigned[38] to the metal-nitrogen ν(M-N) and metal-oxygen ν(M-O) respectively. These new bands were observable only in the spectra of the metal complexes and not in the spectra of its uncomplexed Schiff-base which in turn, confirmed participation of these hetero groups (oxygen of furane and nitrogen of thiadiazole moieties) in the coordination.

Table 2. IR and UV-Visible Spectral Data of the Metal(II) Complexes.

| No | IR ($cm^{-1}$) | $\lambda_{max}$ ($cm^{-1}$) |
|---|---|---|
| 1 | 1615 (s, HC=N), 1585 (s, C=N), 525 (ms, M-N), 455 (ms, M-O) | 26,750, 24,215, 15,475 |
| 2 | 1610 (s, HC=N), 1595 (s, C=N), 525 (ms, M-N), 455 (ms, M-O) | 26,745, 24,315, 15,395 |
| 3 | 1610 (s, HC=N), 1585 (s, C=N), 530 (ms, M-N), 460 (ms, M-O) | 26,810, 24,245, 15,415 |
| 4 | 1610 (s, HC=N), 1595 (s, C = N), 530 (ms, M-N), 460 (ms, M-O) | 26,785, 24,275, 15,450 |
| 5 | 1615 (s, HC=N), 1595 (s, C = N), 525 (ms, M-N), 460 (ms, M-O) | 29,215, 23,725, 13,540, 8,515 |
| 6 | 1610 (s, HC=N), 1585 (s, C=N), 530 (ms, M-N), 455 (ms, M-O) | 29,275, 23,815, 13,775, 8,445 |
| 7 | 1615 (s, HC=N), 1585 (s, C=N), 525 (ms, M-N), 460 (ms, M-O) | 29,545, 23,750, 13,765, 8,470 |
| 8 | 1615 (s, HC=N), 1585 (s, C=N), 530 (ms, M-N), 455 (ms, M-O) | 29,350, 23,805, 13,695, 8,510 |

s=sharp, ms=medium sharp

**Magnetic moment and UV-Visible Spectra**

The UV-Visible spectral bands are recorded in Table 1. The room temperature magnetic moment values of the solid cobalt(II) complexes were found to lie in the range (4.5-4.6 B.M), indicative[39] of three unpaired electrons per Co(II) ion attaining an octahedral environment. Similarly, the Ni(II) complexes showed $\mu_{eff}$ values in the range (3.3-3.5 B.M), corresponding[39] to two unpaired electrons per Ni(II) ion for their ideal six-coordinated configuration.

The electronic spectra of the Co(II) complexes showed three bands observed at 15395-15475, 24215-24275 and 26745-26810 $cm^{-1}$ which may be assigned to ${}^4T_{1g} \rightarrow {}^4T_{2g}(F)$, ${}^4T_{1g} \rightarrow {}^4A_{2g}(F)$ and ${}^4T_{1g} \rightarrow {}^4T_{1g}(P)$ transitions respectively and are suggestive[40] of the octahedral geometry around the cobalt ions.

The Ni(II) complexes exhibited four bands at 8445-8515, 13540-13775, 23725-23815 and 29215-29545 $cm^{-1}$. The first three bands are assigned[40] to the spin-allowed transitions ${}^3A_{2g}(F) \rightarrow {}^3T_{2g}(F)(\nu_1)$, ${}^3A_{2g}(F) \rightarrow {}^3T_{1g}(F)(\nu_2)$ and ${}^3A_{2g}(F) \rightarrow {}^3T_{2g}(P)(\nu_3)$ respectively. The fourth band was of high intensity due to ligand-metal charge-transfer. The occurrence of three spin-allowed transitions supports the octahedral geometry for Ni(II) complexes (Fig 2).

**Antibacterial Properties**

The title Schiff-base and its Co(II) and Ni(II) metal chelates having the same metal ion (cation) but, different anion were evaluated for their antibacterial activity against bacterial species *Escherichia coli (a), Staphylococcus aureus (b) and Pseudomonas aeruginosa (c).* The compounds were tested at a concentration of 30 μg/0.01 mL in DMF solution using the paper disc diffusion method. The susceptibility zones were measured in diameter (mm) and the results are reproduced in Table 3. The susceptibility zones measured were the clear zones around the discs killing the bacteria.

The Schiff-base and its complexes individually exhibited varying degrees of inhibitory effects on the growth of the tested bacterial species. The antibacterial results evidently show that the activity of the Schiff-base became more pronounced when coordinated to the metal ions. The metal ions having different anions had varying antibacterial influence on bacterial species. For example, the Co(II) complex with nitrate anion was more bactericidal than the Co(II) complex with sulfate, oxalate or acetate anions. Similarly, the Cu(II) complex oxalate was more antibacterial than the complex having acetate, chloride or sulfate. Similar results were found in the case of Ni(II) complexes. It was observed that the order of potency, in comparison to the metal complexes having chloride anions evaluated and reported[34] earlier, is as follows,

$$NO_3^- > C_2O_4^{2-} > CH_3CO_2^- > Cl^- > SO_4^{2-}$$

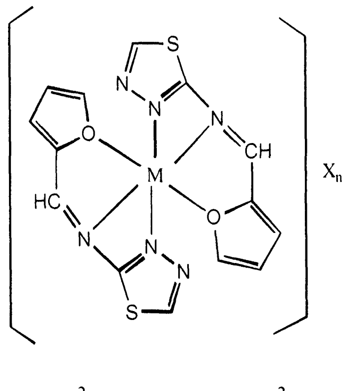

$X = NO_3^-$, $C_2O_4^{2-}$, $CH_3CO_2^-$ or $SO_4^{2-}$, n = 1 or 2

M = Co(II) or Ni(II)

Fig. 2: Proposed Structure of the Metal(II) Complexes (1-8).

On the basis of the above observations, it is claimed that the process of chelation dominantly affects the biological behavior of the compounds that are potent against some bacterial strains. From these studies, it is also proposed that different anions do play a significant role in the biological behavior of the metal chelates. It is suspected that factors such as solubility, different dipole moment and cell permeability mechanisms may be influenced by the presence of the different anions also affect the mechanism of permeation through the lipid layer of the organisms killing more of them effectively.

Table 3. Antibacterial Activity Data of the Schiff base and its Metal Complexes

| Schiff base/ Complex | Microbial Species a | b | c |
|---|---|---|---|
| L | ++ | + | ++ |
| (1) | +++ | +++ | ++ |
| (2) | +++ | ++ | +++ |
| (3) | +++ | +++ | +++ |
| (4) | ++++ | +++ | +++ |
| (5) | +++ | +++ | +++ |
| (6) | ++++ | ++ | +++ |
| (7) | ++++ | +++ | ++++ |
| (8) | +++ | +++ | +++ |

a= *Escherichia coli*, b= *Staphylococcus aureus*,
c= *Pseudomonas aeruginosa*
Inhibition zone diameter mm (% inhibition): +, 6-10 (27-45 %); ++, 10-14 (45-64 %); +++, 14-18 (64-82 %); ++++, 18-22 (82-100 %). Percent inhibition values are relative to inhibition zone (22 mm) of the most active compound with 100 % inhibition.

### ACKNOWLEDGEMENT
The authors gratefully acknowledge Department of Pathology, Qaid-e-Azam Medical College, Bahawalpur, Pakistan in undertaking the antibacterial studies.

### REFERENCES
1. D. R. Williams, "*The Metals of Life*", Van Nostrand, London, (1971).
2. D. H. Brown, W. E. Smith, J. W. Teape and A. J. Lewis, *J. Med. Chem*, **23**, 729 (1980).
3. a) A. Scozzafava, L. Menabuoni, F. Mincione, G. Mincione and C.T. Supuran, *Bioorg. Med. Chem. Lett*, **11**, 575 (2001); b) A. Scozzafava and C. T. Supuran *J. Med. Chem*, **43**, 3677 (2000); c) G. Alzuet, J. Casanova, J. Borras, S. Garcia-Granda, A. Gutierrez-Rodriguez and C. T. Supuran, *Inorganica Chimica Acta*, 273, 334 (1998); d) C.T. Supuran, A. Scozzafava *Eur. J. Med. Chem*, **35**, 867(2000).
4. a) R. J. P. William, "*Quart. Rev*, **24**, 331 (1970); b) R. J. P. William, "*Bioinorganic Chemistry*", American Chemical Society, Washington, (1971).
5. B. Rosenberg and L. V. Camp, *Cancer. Res*, **30**, 1799 (1970)
6. M. J. Clare and J. D. Heeschele, *Bioinorg. Chem*, **2**, 187 (1973).
7. V. L. Narayanan, M. Nasr and K. D. Paull, in "*Tin-Based Antitumor Drugs*", M. Gielen (Ed), NATO ASI Series, Vol. H37, Springer Verlag, Berlin (1990).

8. A. J. Crowe, in "*Metal Based Antitumor Drugs*", M. Gielen (Ed), Vol 1, Freund, London (1988).
9. R. S. Srivastava, *Ind. J. Chem*, **29A**, 1024 (1990).
10. M. J. Seven and L. A. Johnson, "*Metal Binding in Medicine*", Lippincott Co, Philadelphia, PA, 4th Ed (1960).
11. V. K. Patel, A. M. Vasanwola and C. R. Jejurkas, *Ind. J. Chem*, **28A**, 719 (1989).
12. a) A. Scozzafava and C. T. Supuran, *J. Med. Chem*, **43**, 3677 (2000); b) C. T. Supuran, A. Scozzafava, L. Menabuoni, F. Mincione, F. Briganti and G. Mincione, *Metal-Based Drugs*, **6**, 67 (1999); c) C. T. Supuran, F. Mincione, A. Scozzafava, F. Briganti, G. Mincione and M. A. Ilies, Eur. *J. Med. Chem*, **33**, 247 (1998); d) C. T. Supuran, A. Scozzafava, I. Saramet, M. D. Banciu, *J. Enzyme. Inhib*, **13**, 177 (1998); e) C. T. Supuran and A. Scozzafava, *J. Enzyme. Inhib*, **12**, 37 (1997); f) G. Mincione, A. Scozzafava and C. T. Supuran, *Metal-Based Drugs*, **4**, 27 (1997); g) A. Scozzafava and C. T. Supuran, *Metal-Based Drugs*, **4**, 19 (1997).
13. J. M. Pratt, "*Inorganic Chemistry of Vitamin B*", Academic Press, London (1970).
14. S. K. Shapiro and F. Schlenk, "*Transmethylation and Methionine Biosynthesis*", University of Chicago Press, Chicago (1965).
15. J. M. Walsh, *Brit. J. Hospit. Med*, **6**, 45 (1970).
16. E. Ochial, *Coord. Chem. Rev*, **3**, 49 (1968).
17. F. Frieden, *Sci. Amer*, **218**, 102 (1968).
18. R. J. P. William, *Chem. Rev*, **1**, 13 (1968).
19. E. Sinn and C. M. Harris, *Coord. Chem. Rev*, **4**, 391 (1969).
20. M. Barboiu, C. T. Supuran, A. Scozzafava, C. Guran, P. Diaconescu, M. Bojin, V. Iluc and L. Cot, *Metal-Based Drugs*, **6**, 101 (1999).
21. Dich de Vos, P. Clements, S. M. Pyke, D. R. Smyth and E. R. T. Tiekink, *Metal-Based Drugs*, **6**, 31 (1999).
22. M. Geraghly, M. McCann, M. Devereux, F. Cronin, M. Curran and V. McKee, *Metal-Based Drugs*, **6**, 41 (1999).
23. K. Sharma and R. V. Singh, *Metal-Based Drugs*, **7**, 1 (2000).
24. T. Pandey and R. V. Singh, *Metal-Based Drugs*, **7**, 7 (2000).
25. A. Mastrolorenzo and C. T. Supuran, *Metal-Based Drugs*, **7**, 49 (2000).
26. A. Scozzafava, L. Menabuoni, F. Mincione, G. Mincione and C. T. Supuran, *Bioorg. Med. Chem. Lett*, **11**, 575 (2001).
27. C. Walsh, *Science*, 409, 226 (2001).
28. R. V. Singh, *Synth. React. Inorg. Met-Org. Chem*, **16**, 21 (1986).
29. M. A. Mohammad, M. M. El-Enamy and E. L. Basies, *Egypt. J. Pharm. Sci*, **22**, 9 (981).
30. A. M. Osman, K. Hassan, M. E. Kashaf, M. A. Maghraby and M. A. Hassan, *J. Ind. Chem. Soc*, **56**, 521 (1979).
31. B. Dash and M. Patra, *Ind. J. Chem*, **19B**, 894 (1980).
32. V. H. Shah and A. R. Parkash, *Ind. J. Chem*, **54**, 41 (1982).
33. a) Z. H. Chohan and S. K. A. Sherazi, *Synth. React. Inorg. Met.-Org. Chem*, 29, 105 (1999); b) Z. H. Chohan and S. Kausar, *Metal-Based Drugs*, 7, 17 (2000); c) Z. H. Chohan and M. Praveen, *Appl. Organometal. Chem*, 14, 376 (2000) d) Z. H. Chohan and M. Praveen, *Synth. React. Inorg. Met.-Org. Chem*, 30, 175 (2000); e) Z. H. Chohan and M. Praveen, *Appl. Organometal. Chem*, 15, 617 (2001); f) Z. H. Chohan, A. Munawar and C. T. Supuran, *Metal-Based Drugs*, 8, 137 (2001); g) Z. H. Chohan, *Metal-Based Drugs*, 7, 177 (2000); h) Z. H. Chohan, M. A. Farooq and M. S. Iqbal, *Metal-Based Drugs*, **7**, 133 (2000).
34. Z. H. Chohan, H. Pervez, A. Rauf, A. Scozzafava, and C.T. Supuran, *J. Enz. Inhib* (in press).
35. a) A. M. Shallary, M. M. Moustafa and M. M. Bekheit, *J. Inorg. Nucl. Chem*, **41**, 267 (1979). b) W. J. Geary, *Coord. Chem. Rev*, **7**, 81 (1971).
36. L. J. Bellamy, "*The Infrared Spectra of Complex Molecules*", 3rd Ed, Methuen, London, (1966).
37. M. Yongxiang, Z. Zhengzhi, M. Yun and Z. Gang, *Inorg. Chim. Acta*, **165**, 185 (1989).
38. K. Nakamoto, "*Infrared Spectra of Inorganic and Coordination Compounds*", 2nd Ed, Wiley Interscience, New York, (1970).
39. a) B. N. Figgis, "*Introduction to Ligand Fields*", J. Wiley, New York, (1976); b) B. P. Lever, "*Inorganic Electronic Spectroscopy*", Elsevier, Amsterdam, (1984); c) D. Liehr, *J. Phys. Chem*, **67**, 1314 (1967); d) T. M. Dunn, J. Lewis and R. C. Wilkins, "*The Visible and Ultraviolet Spectra of Complex Compounds in Modern Coordination Chemistry*", Interscience, New York, (1960).
40. a) A. B. P. Lever, J. Lewis and R. S. Nyholm, *J. Chem. Soc*, 4761 (1964); b) R. L. Carlin,., "*Transition Metal Chemistry*", Ed, R. L. Carlin, Vol 1, Marcel Decker, New York, (1965); c) D. W. Meek, R. S. Drago and T. S. Piper, *Inorg. Chem.*, **1**, 285 (1962); d) R. S..Drago, "*Physical Methods in Inorganic Chemistry*", Reinhold, New York, (1965); e) B. N. Figgis and J. Lewis, *Prog. Inorg. Chem.*, **6**, 87 (1964).

**Received: March 7, 2002 – Accepted: March 28, 2002 –**
**Accepted in publishable format: March 29, 2002**

